# Intestinal lymphangiectasia in an adult: case report

**DOI:** 10.1590/1677-5449.200160

**Published:** 2021-06-11

**Authors:** Gustavo Sasso Benso Maciel, Brenno Seabra de Mello, Daniel Retsos Loss, Marcelo Soares Quintão, Cleilson Almeida Marchesi, Yasmin de Rezende Beiriz, José Marcelo Corassa

**Affiliations:** 1 Hospital Santa Paula, Vitória, ES, Brasil.; 2 Hospital Santa Casa de Misericórdia de Vitória – HSCMV, Vitória, ES, Brasil.; 3 Escola Superior de Ciências da Santa Casa de Misericórdia de Vitória – EMESCAM, Vitória, ES, Brasil.

**Keywords:** lymphangiectasia intestinal, lymphangiectasia, lymphatic vessels, case report

## Abstract

Intestinal lymphangiectasia is a group of rare diseases characterized by dilation of lymphatic channels. Its pathophysiology comprises obstruction of small bowel lymphatic drainage with secondary dilation of mucosal, submucosal, or subserous lymphatic vessels, distorting villous architecture and causing loss of lymph into the intestinal lumen, leading to malabsorption. The affected lymphatic vessels are primarily located in the small intestine, which is affected to a varying extent. Its etiology is still unknown. The following report presents a rare case of intestinal lymphangiectasia in an adult patient.

## INTRODUCTION

Intestinal lymphangiectasia encompasses a group of rare diseases characterized by dilatation of the lymphatic channels.^1^ It was first described by Waldmann in 1961 and is caused by abnormal intestinal lymphatic drainage which can be congenital, secondary, or idiopathic. Its pathophysiology comprises obstruction of small intestine lymphatic drainage with secondary dilation of mucosal, submucosal, or subserous lymph vessels, distorting the villous architecture and causing loss of lymph into the intestinal lumen, leading to malabsorption.^2^

Ingestion of fats by patients with this pathology results in distension and rupture of the lymph channels, triggering intestinal loss of proteins, lymphocytes, and immunoglobulins.^3^ A combination of hypoalbuminemia, lymphopenia, and hypogammaglobulinemia should trigger a suspicion of this diagnosis.^4^

Affected lymph vessels are primarily located in the small intestine,^5^ which is affected to varying degrees.^1^ The etiology is still unknown. Primary lymphangiectasia is also known as Waldmann’s disease and is generally identified in children before they reach 3 years of age and is occasionally diagnosed in adolescents. When congenital, it is frequently associated with lymphatic abnormalities in any part of the body and also with malformations such as Turner, Noonan, and Klippel-Trenaunay-Weber syndromes.^6^ Theories that have been proposed suggest that lymphatic hypoplasia causes obstruction of lymphatic flow in the intestine.^7^ Several genes have been identified as responsible for lymphogenesis, such as VEGFR3, SOX18, FOXC2, and CCBE1.^8^

Secondary lymphangiectasia is the result of lymphatic obstruction with elevated lymphatic pressure or direct injury to lymphatic channels^1^ from causes such as retroperitoneal fibrosis, chronic pancreatitis, abdominal or retroperitoneal tumors, mesenteric tuberculosis, Crohn’s disease, intestinal malrotation, Whipple’s disease, celiac disease, constrictive pericarditis, and congestive heart failure.^9^

Known treatment options include dietary changes, with addition of medium-chain triglycerides (which are absorbed directly in the portal venous circulation), octreotide, and surgical procedures. Additionally, removal of long-chain fatty acids can reduce lymphatic pressure, averting rupture of the lymph vessels.^10^

An appropriate and definitive treatment for intestinal lymphangiectasia would undoubtedly contribute a new perspective on this disease, which has been studied little. This study was conducted after approval by the Research Ethics Committee (decision number 3.760.871).

## CASE DESCRIPTION

**Figure 1:**
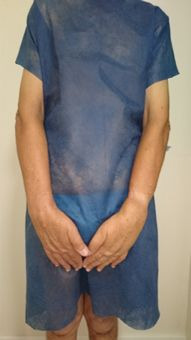
Edema of the upper limbs.

**Figure 2:**
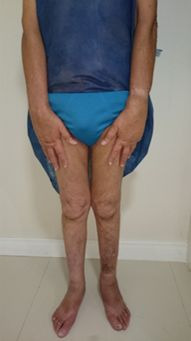
Edema of the lower limbs.

A 58-year-old male patient presented with symmetrical edema of the upper limbs (Figure 1), lower limbs (Figure 2), and scrotum, in conjunction with chronic diarrhea. He weighed 48 kg, with a height of 1.58 m, giving a body mass index (BMI) of 19.2 kg/m^2^, and his primary complaints were paroxysmal nocturnal dyspnea when in decubitus and impaired quality of life because of dyspnea and food intolerance.

The patient had a prior history of congenital edema of the lower limbs and refractory ascites in childhood, associated with diarrhea with food intolerance to any type of diet with lipid components, which still remains. He also had a history of peritoneal tuberculosis that had been treated 30 years previously and an enlarged prostate.

Laboratory tests conducted in June 2019 revealed below normal serum levels of the following elements: zinc, ionic calcium, and magnesium. The patient brought abdominal tomography showing thin and wrinkled jejunal mucosa and prominent Kerckring folds in the ileum, with an appearance suggestive of jejunization of the ileum, bilateral pleural thickening, with costophrenic angle blunting, free liquid in the abdominal cavity, and multiple calcifications distributed throughout the peritoneal cavity, with appearance compatible with remnants of the prior tuberculosis.

**Figure 3:**
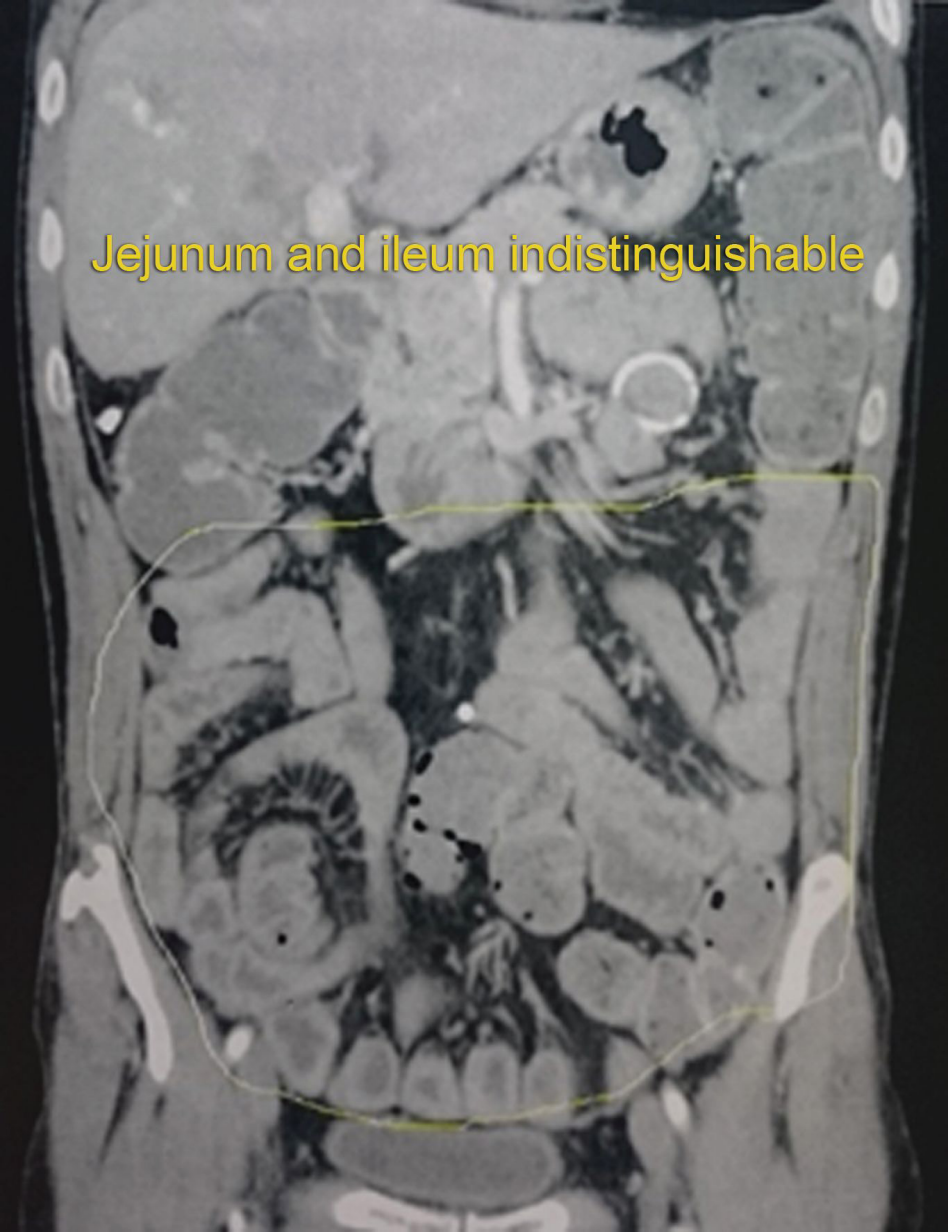
Computed tomography with contrast showing edema of small intestine loops.

**Figure 4:**
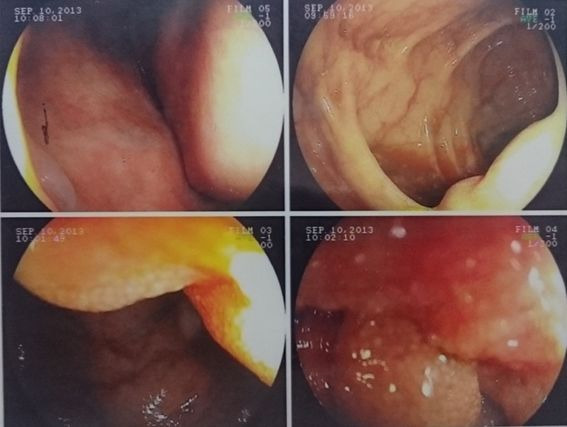
Colonoscopy showing mucosal congestion and lymphatic hyperplasia in the ileum.

Computed tomography with contrast (Figure 3) showed evidence of edema of small intestine loops. Colonoscopy (Figure 4) was able to identify lymphatic congestion in the ascending colon and lymphatic hyperplasia in the ileum.

**Figure 5:**
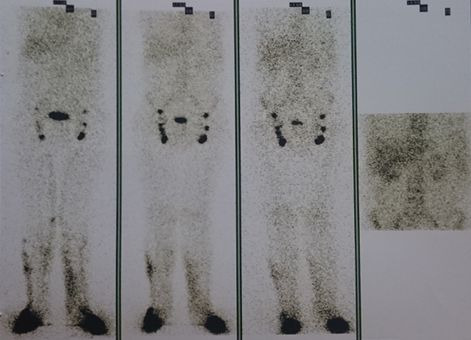
Lymphoscintigraphy showing bilateral dermal backflow.

Lymphoscintigraphy (Figure 5) showed non-obstructive dysfunction of lymphatic drainage of the lower limbs, lateral dermal backflow and rapid ascent of lymph through the lower limbs, but did not identify lymph vessels. Serum tests conducted in August 2019 showed complement, globulins, serology, triglycerides, total and partial cholesterol, and fecal fat tests.

## DISCUSSION

Primary forms are caused by congenital defects affecting formation of lymphatic channels. In turn, secondary forms are caused by another underlying condition, such as radiotherapy, peritoneal infection, abdominal surgery, and others, leading to increased pressure in lymph vessels and loss of lymph into the intestinal lumen. This causes protein losing enteropathies associated with reduced lymphocytes and steatorrhea. There is also malabsorption of chylomicron and liposoluble vitamins.^2^

The consequences of lymph loss include hypoproteinemia, hypoalbuminemia, and lymphopenia, peripheral edema, ascites, pleural effusion, weight loss, stunting, and specific clinical manifestations. The laboratory test results for the patient in the case described are compatible with the literature, since they showed hypoproteinemia, hypoalbuminemia, lymphocytopenia, hypogammaglobulinemia, and reduced transferrin and fibrinogen levels.

As a result of its clinical characteristics, intestinal lymphangiectasia is generally diagnosed by 3 years of age, and female sex and edema are present in 78% of cases.^11^ Although the rarity of presentation of these cases could limit the best therapeutic tactics, the foundations of clinical treatment include a high-protein and low fat diet (with medium-chain lipids), with supplementation of liposoluble vitamins.^11^

Imaging exams for work-up include upper digestive endoscopy with biopsy, colonoscopy with biopsy, and computed tomography with contrast. Histological manifestations are dilatation of lymph vessels, vascular dilatation corresponding to linfangiectasias and moderate chronic duodenitis with villous atrophy. Tomography findings may include speckled or inflammatory pattern in duodenal, jejunal, or ileal mucosa, thick Kerckring folds, thickening of mucosa, and the stack of coins sign.^11^

Administration of albumin is reserved for cases of exacerbation. In turn, octreotide can be used in adult patients who are refractory to clinical treatment with dietary changes, with apparently good results.^11^

In general, surgical treatment with enterectomy may be indicated in limited segmental lesions, because extensive resections can cause short bowel syndrome.^11^

The patient in this case report is in outpatients follow-up with the nutrition and psychiatry teams. He is showing signs of clinical improvement in terms of food tolerance and his general state of anxiety. He has been on a diet based on medium-chain triglycerides. Medium-chain triglycerides are composed of three saturated fatty acids that contain from 6 to 10 carbon atoms and a glycerol molecule. A small number of foods are naturally rich in medium-chain triglycerides, such as human milk, coconut oil, and palm oil. Their structural characteristics enable rapid absorption and rapid bioavailability, because they do not need incorporation into lipoproteins and hepatic transformation. For this reason, they have been employed in treatment of diseases related to intestinal absorption, such as lymphangiectasia, chronic diarrhea, steatorrhea, and celiac disease, for parenteral nutrition, and even for nutritional support in high-performance athletes.
