# Supplementary material for: Intestinal lymphangiectasia in an adult: case report
Source: J Vasc Bras. 2021 Jun 11;20:e20200160. doi: 10.1590/1677-5449.200160 (PMC8210642; doi:10.1590/1677-5449.200160)
Supplement: Translation

# Translation: Linfangiectasia intestinal em adulto: relato de caso

**Authors:** Gustavo Sasso Benso Maciel, Brenno Seabra de Mello, Daniel Retsos Loss, Marcelo Soares Quintão, Cleilson Almeida Marchesi, Yasmin de Rezende Beiriz, José Marcelo Corassa

**Affiliations:** 1 Hospital Santa Paula, Vitória, ES, Brasil.; 2 Hospital Santa Casa de Misericórdia de Vitória – HSCMV, Vitória, ES, Brasil.; 3 Escola Superior de Ciências da Santa Casa de Misericórdia de Vitória – EMESCAM, Vitória, ES, Brasil.

**Keywords:** linfangiectasia intestinal, linfangiectasia, vasos linfáticos, relato de caso

## Abstract

A linfangiectasia intestinal consiste em um grupo de doenças raras caracterizadas pela dilatação dos canais linfáticos. A fisiopatologia compreende a obstrução da drenagem linfática do intestino delgado com dilatação secundária dos vasos linfáticos mucosos, submucosos ou subserosos, que distorcem a arquitetura das vilosidades e conduzem à perda de linfa para a luz intestinal, levando à má absorção. Os vasos linfáticos afetados localizam-se primariamente no intestino delgado, que é atingido em extensão variável. A sua etiologia é ainda desconhecida. O relato a seguir apresenta um raro caso de linfangiectasia intestinal em paciente adulto.

## INTRODUÇÃO

A linfangiectasia intestinal consiste em um grupo de doenças raras caracterizadas pela dilatação dos canais linfáticos^1^. Foi inicialmente descrita por Waldmann em 1961 e é causada por alteração à drenagem linfática intestinal de forma congênita, secundária ou idiopática. A fisiopatologia compreende a obstrução da drenagem linfática do intestino delgado com dilatação secundária dos vasos linfáticos mucosos, submucosos ou subserosos, que distorcem a arquitetura das vilosidades e conduzem à perda de linfa para a luz intestinal, levando à má absorção^2^.

A ingestão de gorduras em pacientes portadores dessa patologia resulta na distensão e ruptura desses canais, originando perda intestinal de proteínas, linfócitos e imunoglobulinas^3^. A associação de hipoalbuminemia, linfopenia e hipogamaglobulinemia deve fazer suspeitar desse diagnóstico^4^.

Os vasos linfáticos afetados localizam-se primariamente no intestino delgado^5^, sendo atingido em uma extensão variável^1^. A sua etiologia é ainda desconhecida. A linfangiectasia primária, também chamada de doença de Waldmann, é geralmente identificada em crianças antes dos 3 anos de idade e, raramente, em adolescentes. Quando congênita, frequentemente está associada a anormalidade linfática em qualquer lugar do organismo, bem como a malformações como a síndrome de Turner, Noonan e Klippel-Trenaunay-Weber^6^. De acordo com teorias propostas, a hipoplasia linfática leva à obstrução do fluxo linfático dos intestino^7^. Vários genes foram identificados como responsáveis pela linfogênese, como VEGFR3, SOX18, FOXC2 e CCBE1^8^.

A linfangiectasia secundária resulta de uma obstrucção linfática com pressão linfática elevada ou lesão direta dos canais linfáticos^1^ por situações tais como fibrose retroperitoneal, pancreatite crônica, tumores abdominais ou retroperitoneais, tuberculose mesentérica, doença de Crohn, má rotação intestinal, doença de Whipple, doença celíaca, pericardite constritiva e insuficiência cardíaca congestiva^9^.

Sabe-se que a modificação da dieta com a inserção de triglicerídeos de cadeia média (os quais são diretamente absorvidos na circulação venosa portal), os ocreotídeos e os procedimentos cirúrgicos foram relatados como opções de tratamento. Além disso, a remoção de ácidos graxos de cadeia longa é capaz de reduzir a pressão linfática, evitando a ruptura dos vasos linfáticos^10^.

Um tratamento adequado e resolutivo para o quadro de linfangiectasia intestinal sem dúvida acrescentaria nova perspectiva para essa doença, que é pouco estudada. O presente trabalho foi realizado após aprovação do Comitê de Ética em Pesquisa (parecer número 3.760.871).

## DESCRIÇÃO DO CASO

Um paciente de 58 anos, sexo masculino, apresentou-se com edema simétrico dos membros superiores (Figura 1), inferiores (Figura 2) e escroto, associado a diarreia crônica. Estava com 48 kg, 1,58 m, índice de massa corporal (IMC) 19,2 kg/m^2^, e as principais queixas eram de dispneia paroxística noturna em decúbito e prejuízo à qualidade de vida devido à dispneia e à intolerância alimentar.

**Figura 1:** Edema de membros superiores.

**Figura 2:** Edema de membros inferiores.

O paciente possui história pregressa de edema dos membros inferiores congênito e ascite refratária na infância, associada a diarreia com intolerância alimentar a quaisquer tipos de dietas com componentes lipídicos a qual persiste até hoje. Além disso, também apresentava histórico de tuberculose peritoneal tratada há 30 anos e de hiperplasia prostática.

Os exames laboratoriais de junho de 2019 apresentavam níveis séricos abaixo do normal dos seguintes elementos: zinco, cálcio iônico e magnésio. O paciente trouxe tomografia abdominal evidenciando pregueamento mucoso jejunal pouco espessado e íleo apresentando válvulas coniventes proeminentes assumindo aspecto de jejunização do íleo, espessamento pleural bilateral com apagamento dos seios costofrênicos, líquido livre na cavidade abdominal e múltiplas calcificações distribuídas em cavidade peritoneal de aspecto residual a tuberculose prévia.

Na tomografia computadorizada (Figura 3) contrastada, pode ser evidenciado edema de alças de intestino delgado. A colonoscopia (Figura 4) permitiu identificar congestão linfática em cólon ascendente e hiperplasia linfática em íleo.

**Figura 3:** Tomografia computadorizada contrastada evidenciando edema de alças de intestino delgado.

**Figura 4:** Colonoscopia identificando congestão mucosa e hiperplasia linfática em íleo.

Na linfocintilografia (Figura 5), pode-se observar disfunção não obstrutiva na drenagem linfática dos membros inferiores, refluxo dérmico lateral e rápida ascensão da linfa via membros inferiores, mas sem identificação de vasos linfáticos. Os exames séricos realizados em agosto de 2019 mostraram complemento, globulinas, sorologias, triglicerídeos, colesterol total e frações e pesquisa de gorduras fecais.

**Figura 5:** Linfocintilografia exibindo refluxo dérmico bilateral.

## DISCUSSÃO

As formas primárias são determinadas por defeitos congênitos na formação dos canais linfáticos. As formas secundárias, por sua vez, são determinadas por outro quadro de base, como radioterapia, infecção peritoneal, cirurgia abdominal, entre outros, o que leva ao aumento da pressão dos vasos linfáticos e à perda de linfa para o lúmen intestinal. Esse quadro causa enteropatia perdedora de proteínas associada a diminuição de linfócitos e esteatorreia. Há ainda má absorção de quilomícrons e vitaminas lipossolúveis^2^.

A perda de linfa tem como consequências hipoproteinemia, hipoalbuminemia e linfopenia, edema periférico, ascite, derrame pleural, emagrecimento, atraso no crescimento e manifestações clínicas específicas. Os achados laboratoriais do paciente do caso relatado corroboram com a referida literatura, visto que apresentava hipoproteinemia, hipoalbuminemia, linfocitopenia, hipogamaglobulinemia, diminuição dos níveis de transferrina e de fibrinogênio.

Em virtude das características clínicas, a linfangiectasia intestinal geralmente é diagnosticada até os 3 anos de idade, sendo gênero feminino e edema presentes em 78% dos casos^11^. Apesar da raridade da apresentação de casos ser capaz de limitar a melhor tática terapêutica, as bases do tratamento clínico incluem dieta hiperproteica e hipolipídica (lipídios de cadeia média), com a inclusão de vitaminas lipossolúveis^11^.

Os exames de imagem complementares incluem endoscopia digestiva alta com biópsia, colonoscopia com biópsia e tomografia computadorizada contrastada. As manifestações histológicas são dilatação de vasos linfáticos, dilatações vasculares correspondentes a linfangiectasias e duodenite crônica moderada com atrofia de vilosidades. À imagem de tomografia, podem ser encontrados padrão espiculado ou inflamatório em mucosa duodenal, jejunal ou ileal, pregas coniventes grossas, espessamento de mucosa e empilhamento de moedas^11^.

A administração de albumina está restrita a quadros de exacerbação. O octreotídeo, por sua vez, pode ser utilizado em casos de adultos refratários ao tratamento clínico com medidas dietéticas, com aparentes bons resultados^11^.

Em linhas gerais, o tratamento cirúrgico com enterectomia está indicado em lesões segmentares limitadas, pois ressecções amplas podem levar à síndrome do intestino curto^11^.

O paciente do caso relatado se encontra em acompanhamento conjunto com equipe de nutrologia e psiquiatria. Além disso, apresenta melhora clínica da aceitação alimentar e quadro geral da ansiedade. Tem sido utilizada uma dieta baseada em triglicerídeos de cadeia média (TCM). Os TCM são compostos por três ácidos graxos saturados, que contêm de 6 a 10 átomos de carbono e uma molécula de glicerol. Poucos alimentos são naturalmente ricos em TCM, como leite humano, óleo de coco e óleo de palma (óleo de dendê). Suas características estruturais permitem rápida absorção e rápida biodisponibilidade por não necessitarem de incorporação às lipoproteínas e de transformação hepática. Por isso, têm sido empregados no tratamento de doenças relacionadas à absorção intestinal, tais como linfangiectasia, diarreia crônica, esteatorreia, doença celíaca, em nutrição parenteral e até suporte nutricional em atletas de alta performance.
